# Changes in pulmonary restrictive parameters by intensive home hemodialysis: a case report

**DOI:** 10.1186/s12882-020-01977-5

**Published:** 2020-08-03

**Authors:** Thatsaphan Srithongkul, Owen D. Lyons, Rose Faratro, Christopher T. Chan

**Affiliations:** 1 Division of Nephrology, University of Toronto, 200 Elizabeth Street, 8N room 846, Toronto, ON M5G 2C4 Canada; 2 University Health Network, Toronto, Canada; 3 Department of Medicine, Siriraj Hospital, Mahidol University, Nakhon Pathom, Thailand; 4 Department of Medicine, Women’s College Hospital, Toronto, ON Canada

**Keywords:** Home hemodialysis, Restrictive lung disorder, Intensive hemodialysis, Case report

## Abstract

**Background:**

Patients with End-Stage Renal Disease (ESRD) are at an increased risk for restrictive lung disease due to accumulation of uremic toxins and volume overload. Hemodialysis is the preferred treatment for improving lung function in dialysis patients. However, the effects of fluid removal and solute clearance by hemodialysis on lung function remain unclear.

**Case presentation:**

We report a case of restrictive lung disorder in a hemodialysis patient, who showed improvement in both clinical and spirometric lung function after initiation of intensive home hemodialysis (32 h per week).

**Conclusion:**

Intensive hemodialysis augments fluid removal and solute clearance, which in turn may improve restrictive lung function.

## Background

Patients with end-stage renal disease (ESRD) commonly present with pulmonary complications related to uremia and volume overload [1]. Kidney failure reduces pulmonary mechanical and ventilatory functions. Restrictive lung defect (defined as percent forced vital capacity (FVC) < 80 with a reduced total lung capacity (TLC)) is the most common pulmonary dysfunction in ESRD and is associated with poor quality of life and clinical outcome [1].

The prevalence of restrictive lung disorder in chronic kidney disease (CKD) patients is 36% and increases to 64% in CKD patients with protein-energy wasting (PEW) [2–4]. Other important risk factors include older age, tobacco smoking, higher body mass index (BMI), and diabetes. Despite the high prevalence of restrictive lung disorder in the CKD population, its pathogenetic mechanisms remain unclear. Several studies have explored the effect of hemodialysis on lung function with mixed results, and longitudinal data are lacking [2, 5, 6].

We report in a patient with restrictive lung disorder and ESRD, who demonstrated gradual improvement in clinical and pulmonary function parameters after conversion from conventional hemodialysis to nocturnal home hemodialysis (4nights per week, 8 h per session).

## Case presentation

A 62-year-old man with ESRD secondary to diabetes presented to Toronto General Hospital with shortness of breath on exertion for 3 years. His co-morbidities include type 2 diabetes, coronary artery disease, gout, hypertension, hyperlipidemia, hypothyroidism, asthma, and obstructive sleep apnea treated by continuous positive airway pressure (CPAP). His mobility was limited by dyspnea (especially with stairs). Given his poor functionality, he was unable to complete any formal exercise program. He denied any chest pain, orthopnea, and paroxysmal nocturnal dyspnea.

He was diagnosed with asthma 10 years ago with pulmonary function testing (PFTs). His pulmonary obstruction was not substantiated by a methacholine challenge. At that time, he presented with shortness of breath on exertion, chest tightness, and occasional cough. He had a remote history of smoking and occasional alcohol use. He was regularly seen in our chronic kidney disease program 3 years prior to the initiation of renal replacement therapy. At that time, PFTs showed a decrease in lung volumes and airflow obstruction with normal diffusive capacity. Based on symptom severity, his inhaler regime included salbutamol (100 mcg) 2 puffs twice daily, ipratropium (17 mcg) 2 puffs twice daily, salmeterol – fluticasone (50/500) 2 puffs twice daily, montelukast 10 mg once daily, ciclesonide (160 mcg) 2 puffs once daily and tiotropium (1.25 mcg) 2 puffs once daily (Table 2).

At the time of hemodialysis initiation, his vital signs were stable, body weight was 99 kg, and there was pronounced bilateral edema. His cardiac examination revealed normal heart sounds with no audible murmurs. There were no abnormal pulmonary findings.

His initial laboratory testing were consistent with ESRD (urea 40 mmol/L, creatinine 775 μmol/L, hemoglobin 107 g/L, ferritin 280 ng/mL, potassium 4.4 mmol/L, bicarbonate 22 mmol/L, calcium 2.27 mmol/L, phosphate 1.93 mmol/L, albumin 36 g/L, intact parathyroid hormone (PTH) 28 pmol/L and an urine protein creatinine ratio 704 mg/mmol).

**Fig. 1:**
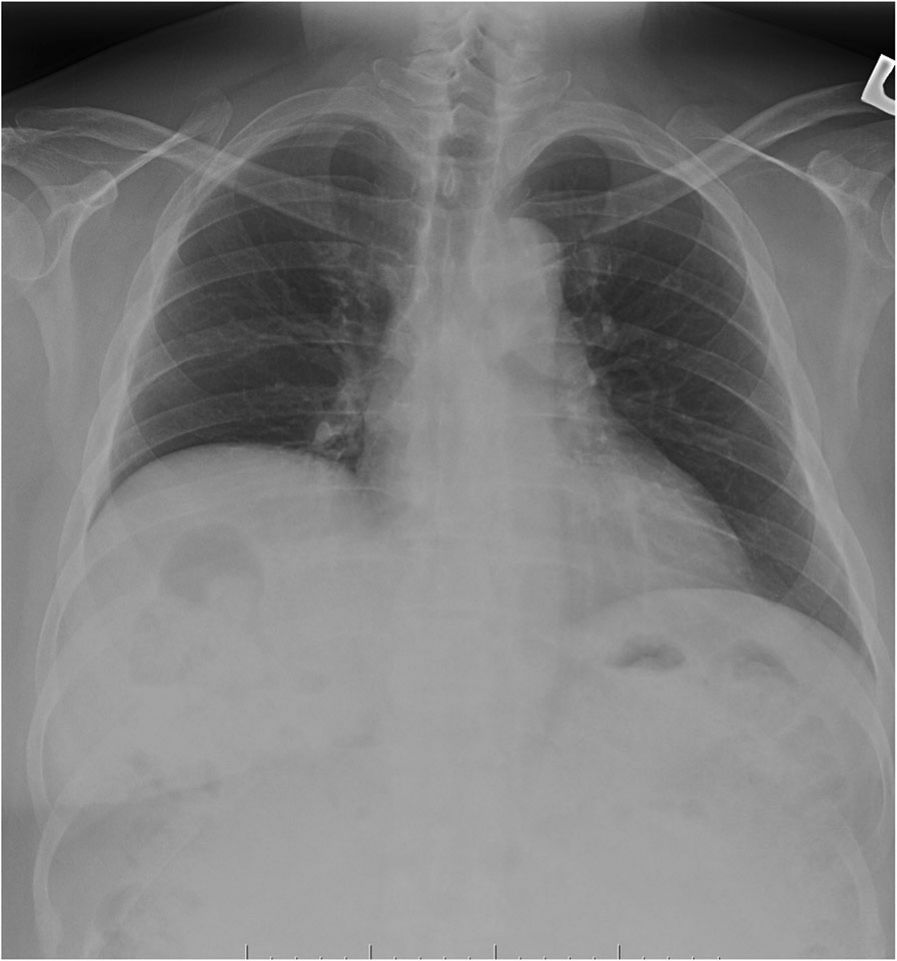
Chest X-ray showed stable mild elevation of the right hemi-diaphragm. There is no lung consolidation or pulmonary edema. The cardio-pericardial silhouette is normal in size

He was initiated on conventional hemodialysis (4 h per session, 3 times a week) in February 2018 due to volume overload. He adhered to his dialysis treatment schedule, and his weight gradually decreased by 7 kg in 3 months. His respiratory symptoms improved, and he was able to exercise using a stationary bicycle without exertional dyspnea. However, he remained dependent on bronchodilators (Table 2). PFTs were repeated in April 2018 (see Table 1). Of note, there was no evidence of airflow limitation. Additional diagnostic tests included a normal electrocardiogram and chest X-ray (Fig. 1). Transthoracic echocardiogram showed an ejection fraction of 55%. Ventricular and atrial assessments showed normal geometry and function bilaterally. CT scan of thorax demonstrated no evidence of interstitial thickening, air trapping, pulmonary edema, or bronchiectasis.

**Table 1:** Pulmonary function test at home hemodialysis initiation and follow up

Parameters	April	September	December
Bodyweight (kg)	91.9	86.5	89.5
BMI (kg/m^2^)	33.8	31.8	32.9
FVC (L)	2.3	2.68	2.72
FVC (%predicted)	59	68	70
FEV_1_ (L)	1.8	2.10	2.03
FEV_1_ (%predicted)	61	70	69
FEV1/FVC	79	78	74.6
Pulse oxymetry (%saturation)	97	97	98
TLC (L)	4.2	4.3	
TLC (% predicted)	70	71	
RV (L)	1.9	1.5	
RV/TLC (%)	45	34	
DLco (ml/min/mmHg)	12.4	14.8	
DLco (% predicted)	68	74	
Corrected DLco (% predicted)	73	74	

*FVC* forced vital capacity, *FEV1* forced expiratory volume in the first second, *TLC* total lung capacity, *RV* residual volume, *TLC* total lung capacity, *DLco* diffusing capacity for carbon monoxide

During 8 weeks of home hemodialysis training, his weight decreased by another 5 kg. The patient’s symptom gradually improved, he denied dyspnea on exertion, or asthmatic attack. His asthmatic medication was tapered as shown in Table 2. He was converted to nocturnal home hemodialysis in June 2018. His hemodialysis prescription was 8 h per session, 4 times a week via left radio-cephalic AV fistula with bicarbonate base dialysate. The remaining dialysis treatment parameters were noted below: Revaclear®; Polyethersulfone membrane, surface area of 1.8 m^2^, urea mass transfer area coefficient (KoA) 1439 mL/min, ultrafiltration coefficient (UFC) 54 ml/hr./mmHg, Blood flow rate (Qb) 350 ml/min and dialysate flow rate (Qd) 500 ml/min.

**Table 2:** Longitudinal follow up of body weight and medication after conversion to home hemodialysis

	Feb	March	April	June	July	Sept	Oct	Dec
**Weight (kg)**	99	96.6	91.9	86.8	85.3	86.5	87.9	89.5
**BMI (kg/m**^**2**^**)**	37.7	36.8	35.01	33.1	32.5	32.9	33.5	32.9
**Ipratropium (17 mcg)** **2 puffs BID**	√	√	√	Hold	Hold	Hold	Hold	Hold
**Tiotropium (1.25 mcg)** **2 puffs OD**	√	√	√	Hold	Hold	Hold	Hold	Hold
**Ciclesonide (160 mcg)** **2 puff OD**	√	Hold	Hold	√	√	Hold	Hold	Hold
**Montelukast 10 mg OD**	√	√	√	Hold	Hold	Hold	Hold	Hold
**Salbutamol (100mcg)** **2 puffs BID**	√	√	√	prn	prn	prn	prn	prn
**Salmeterol – fluticasone (50/500)** **2 puffs BID**	√	√	√	√	√	√	√	Hold

Within 3 months of home hemodialysis, our patient reported further improvement of dyspnea. He indicated that his activities were no longer limited by his respiratory symptoms, and he was able to climb 2 flights of stairs.

His asthma medications were subsequently tapered completely as shown in Table 2.

Routine hemodialysis laboratory data were collected after 3 months of home hemodialysis initiation. His results reflected his intensive hemodialysis prescription (urea 18 mmol/L, creatinine 554 mmol/L, hemoglobin 123 g/L, bicarbonate 23 mmol/L, albumin 41 g/L, calcium 2.29 mmol/L, phosphate 1.35 mmol/L and PTH 37.9 pmol/L.) His urea reduction ratio was 85% with a single-Pool Kt/V of 2.53. Moreover, the patient had a diagnostic methacholine test, and excluded any pulmonary obstruction. His pulmonary function is shown in Table 1.

## Discussions and conclusions

This is the first case report of a patient who had improvements in pulmonary function after conversion to intensive hemodialysis (32 h per week). It is tempting to speculate that enhanced removal of fluid and uremic toxins by augmenting hemodialysis dose may modify pulmonary mechanics and ventilatory function.

Our patient’s respiratory symptoms improved incrementally from the time of initiating hemodialysis to transition to home intensive hemodialysis. It is reasonable to hypothesize that pulmonary interstitial edema and bronchial wall congestion (via salt and water retention) may result in pulmonary restriction. Elevation in alveolar capillary permeability increases hydrostatic pressure of pulmonary vessels. As a result, ventilatory dysfunction within large and small airways may also lead to impaired lung compliance and diffusion defect [1]. Our patient’s initial improvement may be explained primarily by fluid removal, which correlated with a reduction in his body weight. There is a consistent body of literature which associated volume overload with pulmonary dysfunction in ESRD. Yilmaz et al. demonstrated that FEV1 and FVC were significantly lower in dialysis patients with fluid overload. Consequently, increased ultrafiltration volume was independently associated with higher FVC [7]. Similarly, Chase and colleagues demonstrated that increased extravascular lung water, impaired gas exchange and lung compliance was modifiable by enhanced fluid removal [8]. Similarly, Alves et al. demonstrated that sessional ultrafiltration was associated with an acute increase in FEV1 and FVC [9].

After conversion to intensive home hemodialysis, our patient continued to have improvement in lung function without significant changes in his target weight. It is tempting to speculate that other physiologic mechanisms apart from extracellular volume removal may account for the observed changes in lung function. The uremic milieu in ESRD patients may play a role in the pathogenesis of restrictive lung disease. Rahgoshai and colleagues demonstrated an increase in FVC after hemodialysis without significant changes in target weight [10]. Similarly, Navari et al. reported a significant increase of FVC and FEV_1_ in patients using bicarbonate based hemodialysis independent of ultrafiltration [11]. It was postulated that the uremic milieu may impair respiratory muscles leading to a reduced vital capacity in dialysis patients [12–14]. It is also plausible that chronic inflammation and malnutrition caused by uremia [15] may compound the severity of restrictive lung disease [3]. Intensive hemodialysis may modulate inflammatory cytokines levels. Indeed, in another observational study, reduction in endothelin-1 is associated with amelioration bronchoconstriction and pulmonary hypertension in dialysis patients [16]. Unfortunately, we did not measure inflammatory markers in our patient, which warrants further prospective examination.

In summary, restrictive lung disorder is a common complication in the ESRD population. We reported gradual improvement in lung function in a patient undergoing intensive hemodialysis. Our case suggests that chronic accumulation of uremic toxins and volume overload may contribute to the pathogenesis of restrictive lung disorder in dialysis patients. Conversion from conventional hemodialysis to intensive hemodialysis augments fluid removal and solute clearance which may represent a viable option in the treatment of restrictive lung dysfunction in patients with ESRD.

## Data Availability

Further clinical data of this case are available from the corresponding author upon reasonable request.

## Abbreviations

ESRD: End-stage renal disease
CKD: chronic kidney disease
PFTs: pulmonary function testing
FVC: Forced vital capacity
FEV1: Forced expiratory volume in the first second
TLC: Total lung capacity
RV: Residual volume
DLco: Diffusing capacity for carbon monoxide
